# Pangenome analysis of Tanzanian clinical *Klebsiella pneumoniae* reveals pandemic clones with high genome plasticity and versatile mobilome, virulome, and resistome profiles

**DOI:** 10.1128/spectrum.01947-25

**Published:** 2025-10-20

**Authors:** Samweli Y. Bahati, Reuben S. Maghembe

**Affiliations:** 1 Department of Microbiology and Parasitology, Faculty of Medicine, St. Francis University College of Health and Allied Sciences (SFUCHAS) 518753, Ifakara, Tanzania; 2 Department of Data Science and Analytics, Kibong’oto Infectious Disease Hospital (KIDH), Hai, Kilimanjaro, Tanzania; 3 Department of Genomics and Bioinformatics, AfroBiomics Co. Ltd., Dar es Salaam, Tanzania; Nanchang University, Nanchang, Jiangxi, China

**Keywords:** *Klebsiella pneumoniae*, pangenome, resistome, mobilome, virulome, Tanzania

## Abstract

*Klebsiella pneumoniae* is a rapidly evolving pathogen with a diverse pangenome whose mobilome and resistome remain elusive. Here, we aimed to delineate the pangenome of 198 isolates from Tanzanian regions. Raw Illumina reads retrieved from public repositories were assembled and analyzed using multilocus sequence typing, core-genome single nucleotide polymorphism (SNP)-based phylogeny, and capsular polysaccharide (K-locus) and lipopolysaccharide O-antigen (O-locus) typing. A total of 184 isolates were classified as *Klebsiella pneumoniae sensu stricto*, while 14 belonged to other species within the *Klebsiella pneumoniae* complex. We identified 90 sequence types (STs), including global high-risk ST45, ST39, ST336, ST14, ST1552, and ST17. KL24 and KL25 were the most common K-loci, while OL2α.1 and OL2α.2 were dominant O-loci. Pangenome analysis revealed 30,992 gene families, distributed as persistent (13.6%), shell (11.2%), and cloud (75.2%) genes, suggesting an open pangenome structure. Core-genome SNP-based phylogeny confirmed clonal expansion and lineage clustering. Virulence profiling showed yersiniabactin in 44% of isolates. Most genomes carried key fimbrial and iron uptake genes. Resistome analysis revealed near-universal presence of *bla_CTX-M-15*, *oqxA/B*, *fosA6*, *sul2*, and *marA*. Plasmid typing identified IncF-type (76%) and Col-type (54%) plasmids, while over 120 mobile genetic elements were detected, whose frequencies were on a huge spectrum of insertion sequences (e.g., IS5075 and MITE*Ype1*) and transposons (e.g., Tn5403 and Tn6082). Conclusively, Tanzanian *K. pneumoniae* strains exhibit extensive genomic plasticity, high-risk lineages, and a versatile mobilome, calling for national genomic surveillance to inform intervention strategies.

**IMPORTANCE:**

The *Klebsiella pneumoniae* complex comprises a diverse group of bacterial pathogens adapted to thrive over a wide range of environments. Isolates from clinical and environmental samples are implicated in nosocomial infections and multidrug resistance, with similar genome structures and inherent genes. Our study presents the first pangenome report underlying genomic plasticity of *K. pneumoniae* isolates from Tanzanian clinical specimens, demonstrating versatile clones, mobilome, and resistome profiles. Combining these profiles with the versatility of K and O structures, our study emphasizes the need for comprehensive multidisciplinary surveillance studies to optimize therapeutic and vaccine development.

## INTRODUCTION

*Klebsiella pneumoniae* is a common gram-negative pathogen responsible for a wide range of clinical infections, including urinary tract infections, pneumonia, sepsis, and wound infections. It is particularly associated with hospital-acquired infections in neonates and immunocompromised individuals. The clinical success of *K. pneumoniae* is largely driven by its genomic plasticity, which facilitates the acquisition and dissemination of antimicrobial resistance (AMR) determinants via plasmids and other mobile genetic elements (MGEs) (1). Of particular concern is the global emergence of extended-spectrum β-lactamase (ESBL)-producing *K. pneumoniae*, frequently associated with the *^bla^CTX-M-15* gene, which confers resistance to third-generation cephalosporins, complicating therapeutic options (2). In Tanzania, ESBL-producing *K. pneumoniae* has been increasingly reported across hospital and community settings, reflecting its widespread and poorly contained transmission (3–5). A genomic surveillance study in Dar es Salaam identified a conserved IncFIIK5/IncR plasmid carrying *^bla^CTX-M-15* in over 70% of isolates from pediatric patients, spanning genetically diverse lineages, suggesting horizontal dissemination (5). A study from a neonatal unit in Mwanza linked ESBL-producing *K. pneumoniae* (predominantly ST45) to severe neonatal sepsis and high mortality (3). More recently, fecal carriage of ESBL-producing Enterobacterales, mainly *Escherichia coli* and *K. pneumoniae*, was reported in 70.9% of patients with urinary tract infections in rural Tanzania (4). Despite these findings, most studies have relied on phenotypic resistance testing or targeted polymerase chain reaction (6–8), providing a limited scope of the genomic context and its public health implications. Genomic frameworks are crucial for understanding the transmission dynamics, evolutionary pressures, and the dissemination of multidrug-resistant (MDR) lineages in low-income and middle-income countries (1).

In this study, we aimed to delineate the pangenome of the currently available data sets, to extrapolate our understanding of the genetic complexity of circulating strains in Tanzania. Information from this comprehensive analysis aids in uncovering the patterns and dynamics of transmission of virulence and antibiotic resistance in Tanzania.

Utilizing multilocus sequence typing (MLST), core-genome phylogeny, AMR and virulence gene profiling, capsule and O-antigen serotyping, and mobilome analyses, we report a complex open pangenome, a spectrum of MGEs and versatile plasmids, collectively accounting for untapped virulome and resistome profiles.

## MATERIALS AND METHODS

### Data collection and metadata curation

In this study, we analyzed a total of 198 *Klebsiella pneumoniae* whole-genome sequencing data sets obtained from the NCBI Sequence Read Archive under the following BioProject accession numbers: PRJEB65607, PRJNA951629, PRJEB20875, and PRJNA503964. These isolates were collected from diverse clinical and geographic settings across three regions of Tanzania, offering a broader perspective than any single study alone. Specifically, the data sets included stool samples from children with diarrhea collected in Dar es Salaam between August 2010 and July 2011 (5), stool samples from patients with urinary tract infections in 2021 (4), rectal swabs from orthopedic patients in Mwanza collected in 2020, and blood samples from neonatal sepsis cases in Mwanza collected in 2016 (3). Associated metadata, including sample year, geographic origin, clinical source, and disease condition, were systematically curated from BioSample records and the published literature to ensure completeness and accuracy. This comprehensive metadata curation allowed for a contextually informed comparative genomic analysis of *K. pneumoniae* across multiple Tanzanian healthcare and community settings.

### Quality control and genome assembly

Raw Illumina reads were quality-checked using FastQC (v0.11.9). Adapter sequences and low-quality bases (Phred score <20) were trimmed using Trimmomatic (v0.39). High-quality reads were assembled *de novo* using SPAdes (v3.15.4) with default parameters. Assembly quality was assessed using QUAST (v5.2.0); metrics including N50, genome size, guanine-cytosine (GC) content, and total contig count were recorded. Assemblies with >500 contigs or <90% completeness were excluded from further analysis.

### Species confirmation and typing

Species-level identification was performed using Kleborate (v2.3.0) (9). MLST was assigned using Kleborate’s inbuilt PubMLST scheme.

### Genome annotation

Genome assemblies were annotated using Bakta (v1.7.0) (10), with species-specific settings for *Klebsiella pneumoniae*. Annotations included coding sequences, tRNAs, rRNAs, ncRNAs, and pseudogenes. Functional annotation was assigned using curated databases (UniRef90, Pfam, TIGRFAMs), enabling downstream integration of gene product names, COG categories, Gene Ontology terms, and EC numbers. The resulting GFF3 files were used for pangenome and comparative analyses.

### Pangenome analysis

Pangenome profiling was performed using PPanGGOLiN (v1.2.74) (11). Annotated GFF3 files from Bakta were input to cluster genes based on ≥80% amino acid identity. Gene families were partitioned into persistent (core), shell (accessory), and cloud (unique) components using a probabilistic graph model. A tile plot and a U-shaped frequency plot were used to visualize gene distribution and genome plasticity. Pie charts were generated to illustrate the composition of gene partitions and functional modules.

### SNP calling and phylogenetic analysis

Core-genome single nucleotide polymorphisms (SNPs) were identified using Snippy (v4.6.0) with *K. pneumoniae* MGH 78578 (GCF_000016305.1) as reference. SNPs shared by ≥95% of isolates were retained using Snippy-core. A maximum-likelihood phylogenetic tree was inferred from the core SNP alignment using IQ-TREE (v2.2.0). The best-fitting model was selected via ModelFinder using Bayesian Information Criterion, and ultrafast bootstrapping (1,000 replicates) assessed branch support.

### Virulome and resistome analysis

Virulence gene detection and scoring were performed using Kleborate (v2.3.0), screening for loci such as *yersiniabactin (ybt*), *aerobactin (iuc*), *salmochelin (iro*), *colibactin (clb*), and *rmpA/rmpA2*. Additional virulence gene detection was performed using ABRicate (v1.0.1) with the VFDB database (≥90% identity, ≥80% coverage). AMR genes were identified using ABRicate with ResFinder and CARD databases under the same thresholds. Resistance scores were computed following Kleborate scoring criteria. Serotyping of O-antigen and capsular (K) loci was conducted using Kaptive via Kleborate. To ascertain hypermucoviscosity-related hypervirulence, FASTA-format assembly contigs of unique isolates were confirmed by reanalysis using the K-PAM platform (12).

### Plasmid replicon and mobile genetic element profiling

Plasmid replicons were identified using ABRicate with the PlasmidFinder database (https://github.com/genomicepidemiology/plasmidfinder), using thresholds of ≥90% identity and ≥80% coverage. MGEs, including insertion sequences (ISs), transposases, and integrative elements, were identified using MobileElementFinder (MEFinder v1.0.3). Presence/absence matrices were used to visualize the distribution of MGEs, plasmids, AMR genes, and virulence genes across the data set using heatmaps.

## RESULTS

### Data set overview and genome quality metrics

A total of 198 *Klebsiella* isolates from diverse clinical studies conducted across three Tanzanian regions were retrieved and processed for comparative genomic analysis. Raw sequencing reads revealed high-quality data with a median read length of 151 bp and an average of 160.7 bp (range: 110–246 bp). The average GC content was 56.78% (range: 51%–69%), and the mean sequence duplication level was 27.86% (range: 0.08–0.52). The average assembly length was 5.57 Mbp, with a mean N50 of 155.25 kbp and a maximum contig size of 400.42 kbp. Assemblies with >500 contigs or <90% completeness were excluded. After filtering, all 198 assemblies met quality thresholds and were included in downstream analyses. Full assembly statistics, quality control, and sample metadata details are available in the Supplementary Material.

### Species confirmation and sequence type diversity

Genome-based species identification confirmed that 184 of the 198 isolates belonged to *Klebsiella pneumoniae sensu stricto*. The remaining 14 isolates were classified as other members of the *K. pneumoniae* species complex (KpSC), comprising 10 *K*. *quasipneumoniae* subsp. *quasipneumoniae*, 3 *K*. *variicola* subsp. *variicola*, and 1 *K*. *quasivariicola*. MLST analysis of 198 isolates revealed substantial genetic diversity, with a total of 90 distinct sequence types (STs) identified. The most prevalent ST was ST45 (*n* = 21), followed by ST39 and ST336 (*n* = 9 each), ST14 and ST348 (*n* = 6 each), and ST1552 and ST17 (*n* = 5 each). Additional frequently detected STs included ST35, ST37, and ST391 (*n* = 4 each), while ST15, ST405, ST111, ST48, ST30, ST323, ST13, ST661, ST307, and ST834 were each identified in three isolates (*n* = 3 each). Seventeen STs occurred in two isolates each, including ST280, ST471, ST1726, ST788, ST268, ST25, ST394, ST76, ST101, ST367, ST367-2LV, ST3403, ST397, ST3405, ST29, ST3433, and ST540. Notably, 67 isolates represented unique STs (singletons). Details are provided in the Supplementary Material.

### Pangenome architecture

**Fig 1:**
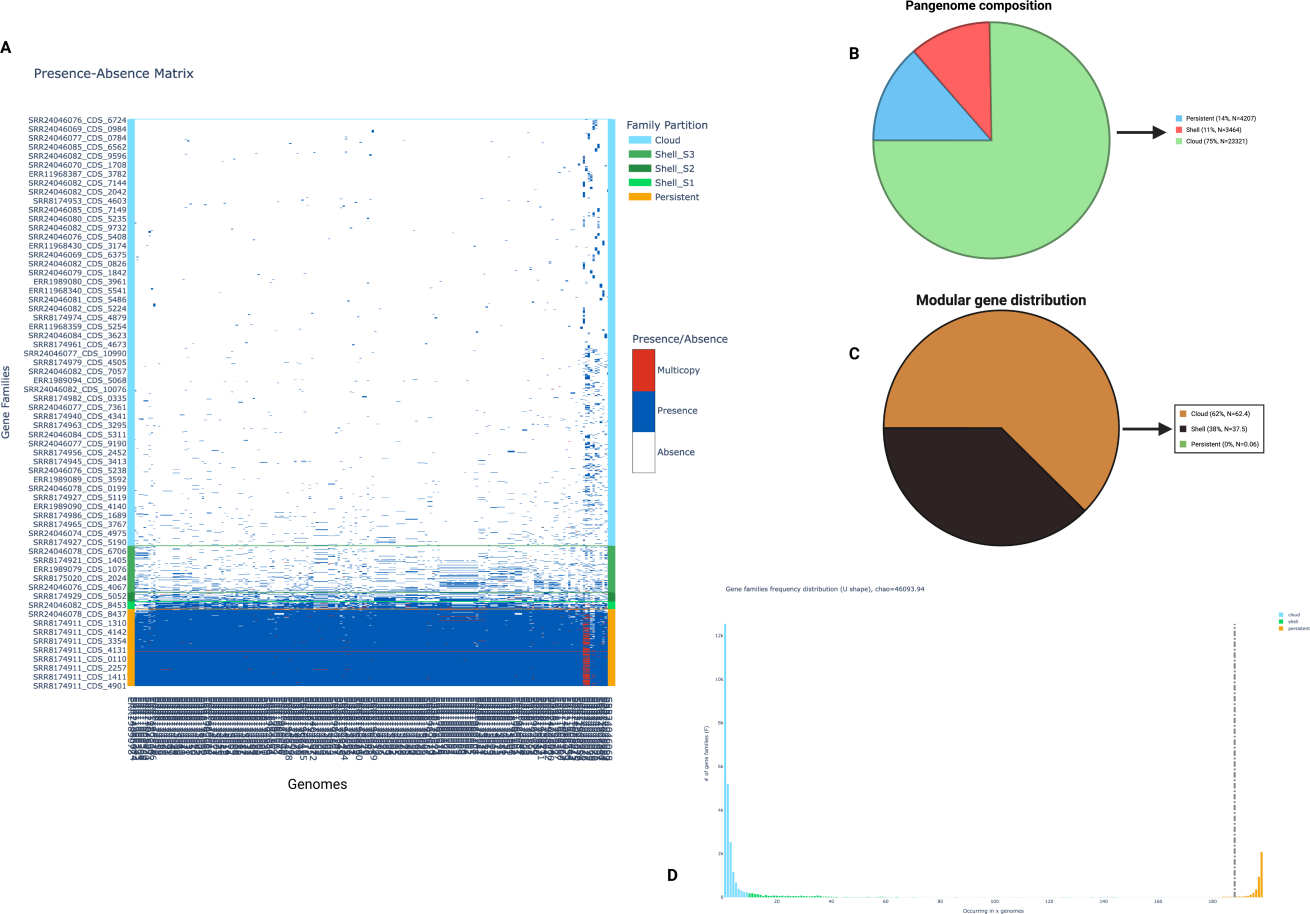
Comparative pangenome composition. (**A**) Gene presence–absence matrix across all isolates, with hierarchical clustering highlighting distinct genomic profiles and patterns of accessory gene distribution. The matrix illustrates the total pangenome, partitioned into the core genome (persistent genes; orange), accessory genome (shell genes S1–S3; green), and strain-specific unique genes (cloud genes; blue). Gene copy number and gene family presence are indicated: red blocks represent multicopy genes, blue blocks indicate the presence of a gene family, and white blocks indicate gene family absence. (**B**) Pangenome composition illustrated as a pie chart, depicting the relative proportions of persistent (blue), shell (red), and cloud (green) gene categories across the data set. (**C**) Modular distribution of genes within the pangenome, with persistent genes shown in green, shell genes in black, and cloud genes in brown, highlights the genome’s structural dynamics. (**D**) The rarefaction curve demonstrates the openness of the genomes, as evidenced by the continual discovery of novel genes with the inclusion of additional genomes.

Pangenome analysis of 198 *Klebsiella pneumoniae* genomes revealed a total of 1,045,769 genes, grouped into 30,992 gene families. A total of 4,207 gene families (13.6%) were categorized as persistent, present in ≥85% of genomes. The shell genome comprised 3,464 gene families (11.2%), subdivided into shell-S1 (*n* = 413), shell-S2 (*n* = 506), and shell-S3 (*n* = 2,545), while the cloud genome, representing rare genes found in <5% of genomes, accounted for 23,321 gene families (75.2%) (Fig. 1B). Gene frequencies ranged from 0.01 to 1.0, with standard deviations of 0.21 for shell genes and 0.01 for cloud genes.

The gene presence/absence matrix illustrated the variable distribution of gene partitions persistent, shell, and cloud across genomes (Fig. 1A). Additionally, 879 gene modules were identified, encompassing 4,646 gene families, of which 62.4% belonged to the cloud genome, 37.5% to the shell genome, and only 0.06% to the persistent genome (Fig. 1C). The gene frequency distribution exhibited a U-shaped curve, indicating a high proportion of genes present in either nearly all or very few genomes, characteristic of an open pangenome structure (Fig. 1D).

### SNP-based phylogeny and genomic clustering

**Fig 2:**
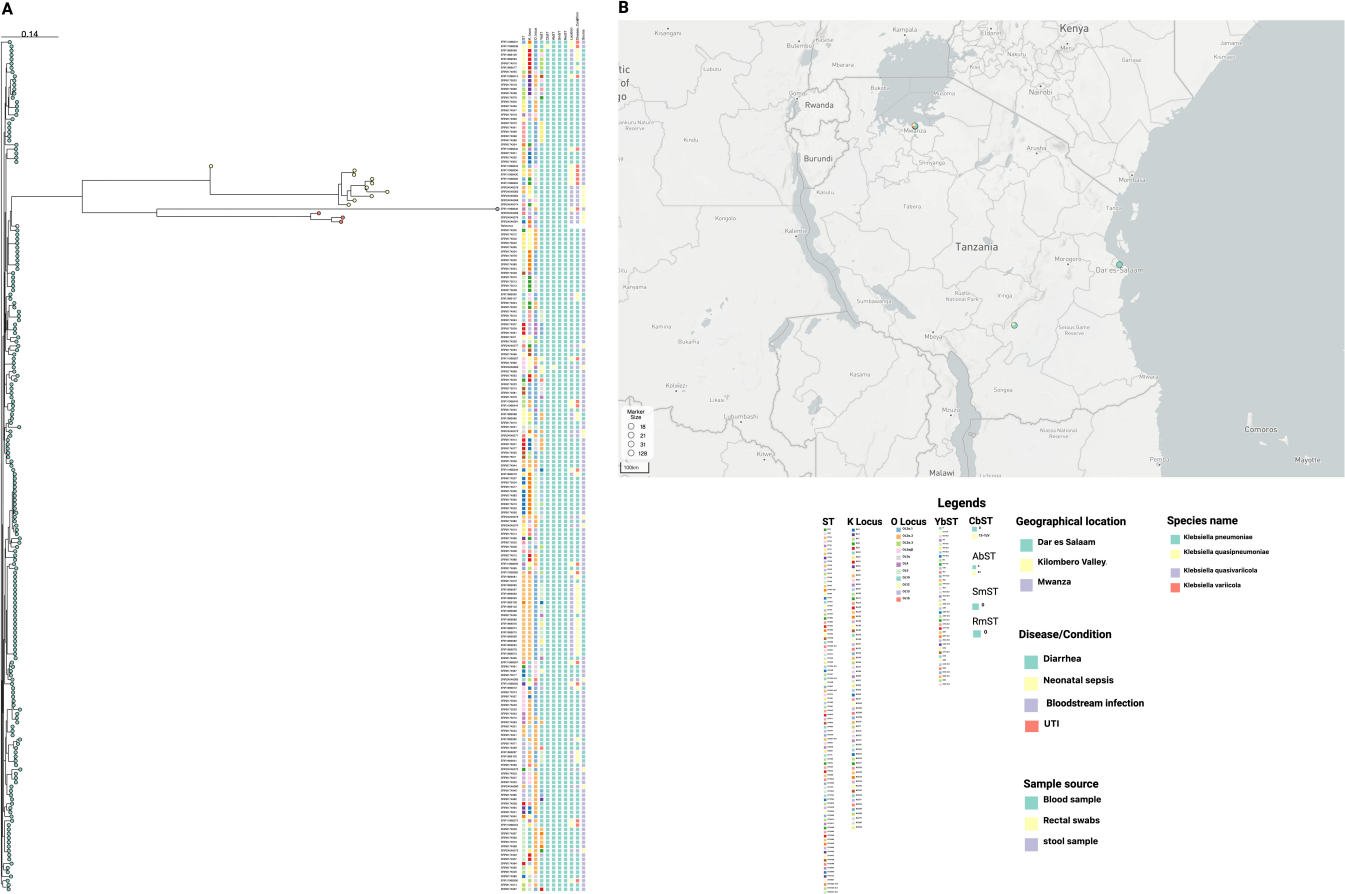
Phylogenomic analysis and geographic distribution of *Klebsiella* clinical isolates from Tanzania. (**A**) Phylogenomic tree based on core-genome SNP alignments showing the population structure and genetic diversity of the isolates. Each leaf represents a single genome, color-coded by species: *K. quasipneumoniae* (yellow), *K. pneumoniae* (mint green), *K. variicola* (red), and *K. quasivariicola* (purple). Isolates are annotated with multilocus STs, capsular loci (K-locus), O-antigen loci (O-locus), and virulence-associated subtypes including yersiniabactin (YbST), colibactin (CbST), aerobactin (AbST), salmochelin (SmST), and *rmpA* (RmST). Metadata tracks show geographic origin, clinical condition, and specimen source (blood, rectal swab, or stool). (**B**) Map of Tanzania showing sampling locations (Dar es Salaam, Morogoro, and Mwanza). Markers indicate the number of isolates recovered at each site. A color-coded legend defines the categories for species, virulence factors, and metadata annotations.

A total of 683,177 high-quality core-genome SNPs were identified among the 198 genomes. The annotated tree in Fig. 2A represents multilocus STs, capsular loci (K-locus), O-antigen loci (O-locus), yersiniabactin sequence types (YbST), colibactin (CbST), aerobactin (AbST), salmochelin (KSmST), rmpA (RmST), geographical location, specimen type, and disease condition metadata. STs such as ST45, ST39, ST336, and ST14 were observed to cluster together. Similarly, isolates sharing the same K-locus and O-locus types were also grouped within the same clades. The distribution of isolates by country and sampling sites was presented as a geographic map (Fig. 2B).

### Virulence gene distribution

**Fig 3:**
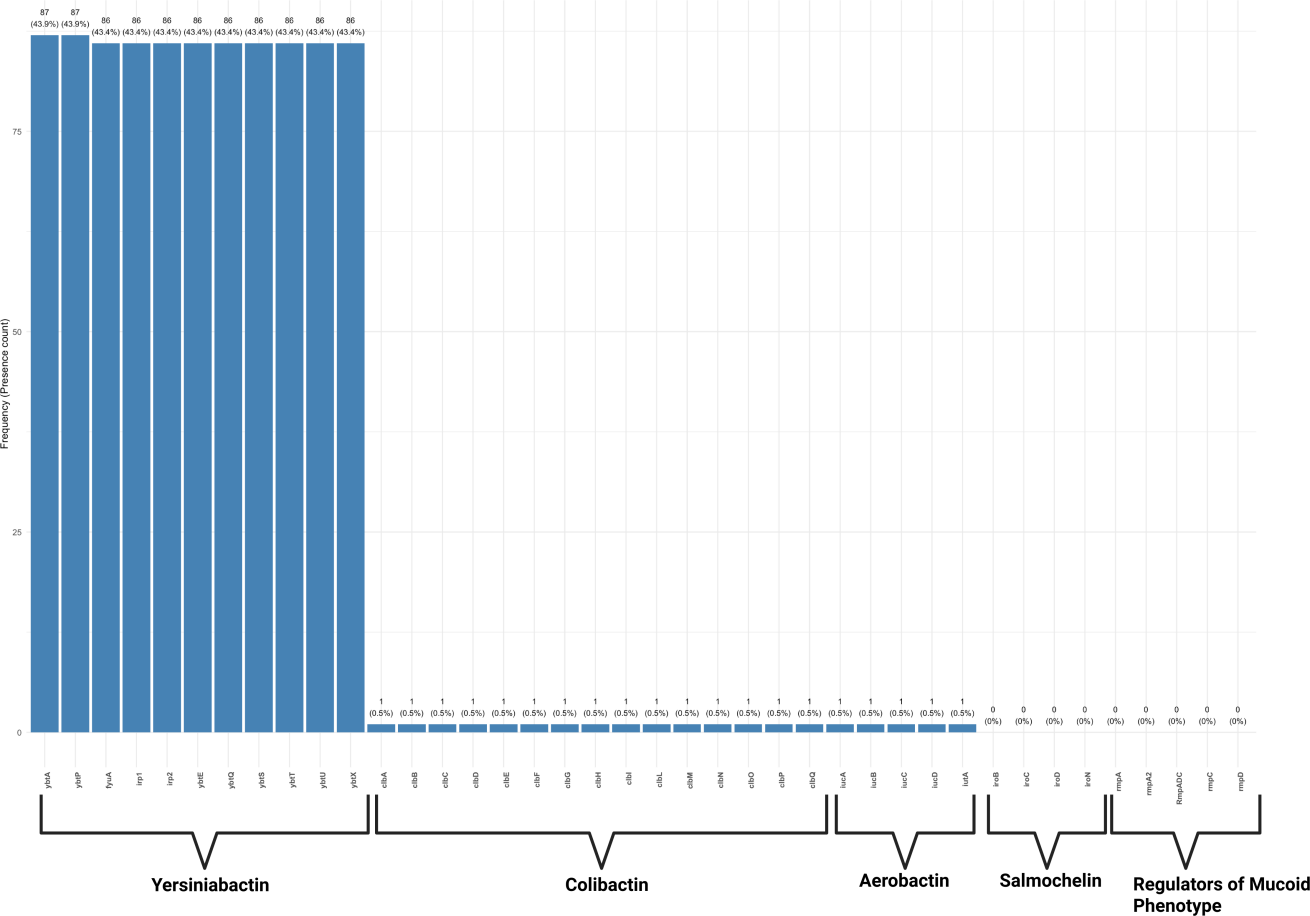
Distribution of siderophore gene clusters among the isolates. The bar plot shows the proportion (%) and count of genes encoding siderophore gene clusters, including yersiniabactin, colibactin, aerobactin, salmochelin, and regulators of the mucoid phenotype. The data were generated using Kleborate, with blue bars representing the percentage of isolates carrying each gene cluster.

Virulence gene profiling using Kleborate identified *ybtA* and *ybtP* in 87 of 198 isolates (43.94%), while *fyuA*, *irp2*, *ybtE*, *ybtQ*, *ybtS*, *ybtT*, *ybtU*, and *ybtX* were each detected in 86 isolates (43.43%). Notably, the operons *clbABCDEFGILMNPQ* (colibactin), *iucABCD-iutA* (aerobactin), and *iroBCDN* (salmochelin) were present in only one isolate (0.51%), specifically ERR11968338 (ST348). The regulators *rmpA* and *rmpA2* were absent in all genomes analyzed. These findings are summarized in the virulence gene frequency (Fig. 3).

**Fig 4:**
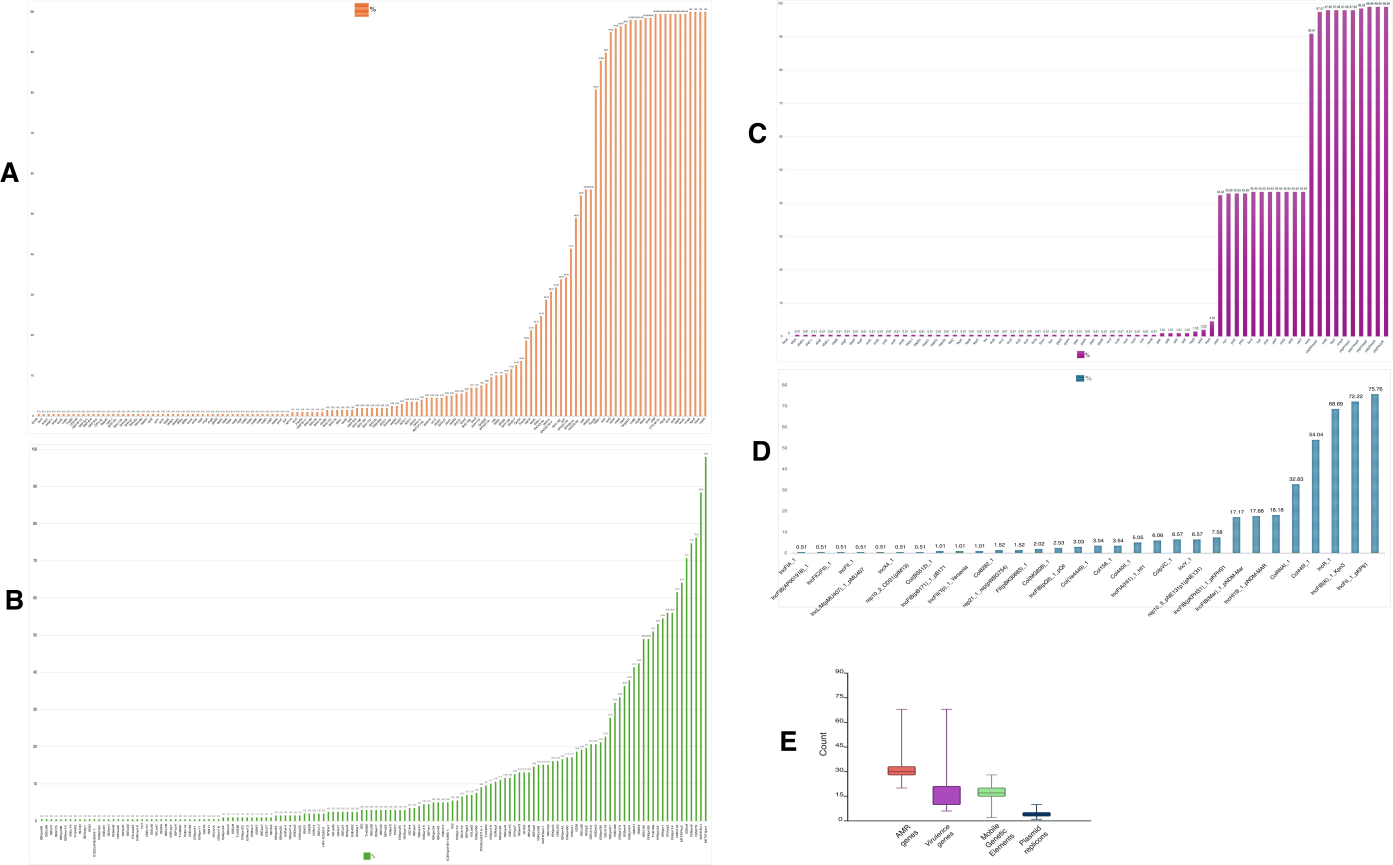
Summary of virulence genes, MGEs, plasmid replicons, and AMR determinants. (**A**) Bar plot showing the prevalence (%) of acquired AMR genes, including β-lactamases, efflux pump genes, and other resistance determinants. (**B**) Bar plot showing the prevalence (%) of MGEs, including insertion sequences and integrative elements. (**C**) Bar plot showing the prevalence (%) of key virulence genes.(**D**) Bar plot showing the frequency (%) of plasmid replicon types, including IncF, IncR, and Col-type replicons. (**E**) Boxplots showing the distribution (median and interquartile range) of the total number of detected features per isolate for virulence genes, AMR genes, MGEs, and plasmid replicons.

**Fig 5:**
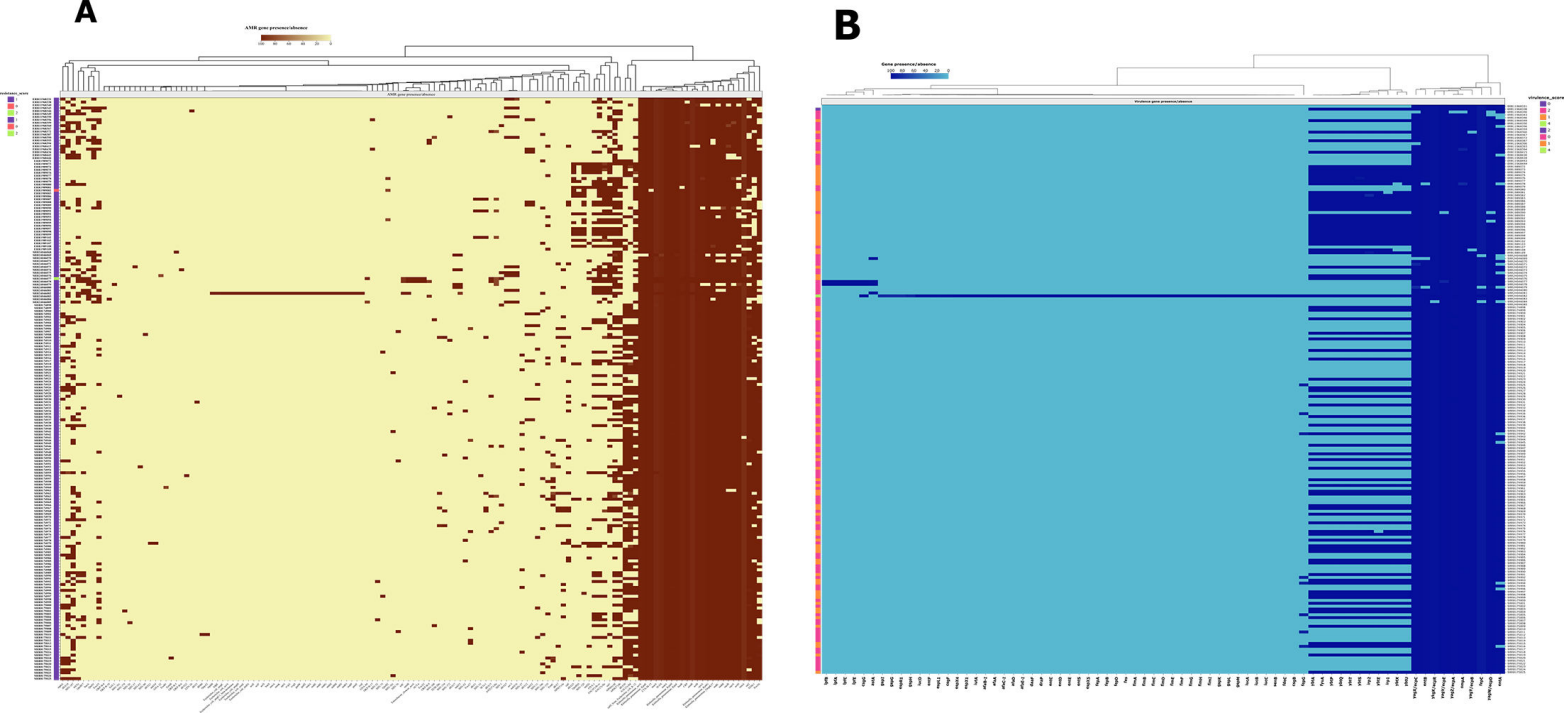
Gene profiling of virulence and AMR genes with corresponding virulence and resistance scores. (**A**) Heatmap showing the presence or absence of AMR genes, clustered by gene presence patterns across isolates. Gene detection was performed using ABRicate (CARD database). The heatmap color scale represents percent identity: yellow indicates absence or lower identity matches; dark red indicates gene presence at higher identity. Kleborate resistance scores are indicated: 0 (red), 1 (purple), and 2 (green). (**B**) Heatmap showing the presence or absence of virulence genes, clustered by similarity in virulence gene profiles. Gene detection was performed using ABRicate (VFDB database). The heatmap color scale represents percent identity: sky blue indicates absence or lower identity; navy blue indicates gene presence at higher identity. Kleborate virulence scores are indicated: 0 (purple), 1 (orange), 2 (pink), and 4 (green). Gene presence was defined as ≥90% sequence identity and ≥80% coverage. Clustering was performed using hierarchical clustering with Euclidean distance.

Overall, the number of virulence genes per isolate ranged from 6 to 68 (Fig. 4E). The most prevalent virulence determinants included *entB*, *fepC*, and *ompA*, each found in 194 isolates (97.98%), as well as *yagV/ecpE*, *yagZ/ecpA*, and *ykgK/ecpR*, each present in 196 isolates (98.99%). *yagY/ecpB* was found in 195 isolates (98.48%). The isolate SRR24046082 (ST307) showed the highest virulence score of 4, while ERR11968338 (ST348) scored 2 despite harboring multiple virulence loci. Most isolates had a virulence score of 0 or 1 (see Fig. 5B). Conversely, low-frequency genes such *as afaA*, *afaB-I*, *afaC-I*, *afaD*, *afaE-I*, *draP*, *fimH*, *iucA*, and *espX5* were each detected in only one isolate (0.51%), while *fimA* was absent (Fig. 4C). The core occurrence and absence of these virulence genes across all *Klebsiella* isolates are depicted in Fig. 5B.

### Resistome profile

A total of 6–68 AMR genes were detected per isolate (Fig. 4E). Frequently detected genes included *CTX-M-15* (197/198, 99.49%), *TEM-1* (174/198, 87.88%), *FosA6* (160/198, 80.81%)**,** *sul2* (178/198, 89.9%)**,** *oqxA* (192/198, 96.97%)**,** and *oqxB* (194/198, 97.98%). Other highly prevalent genes were *acrB* (197/198, 99.49%)**,** *KpnF* (198/198, 100%)**,** *marA* (198/198, 100%)**,** and *msbA* (198/198, 100%). Among aminoglycoside-modifying enzymes, *AAC(3)-IId* was detected in 108 isolates (54.55%)**,** *APH(3″)-Ib* in 82 (41.41%)**,** and *APH(6)-Id* in 97 (48.99%). Fluoroquinolone resistance determinants such as *AAC(6″)-Ib-cr* were identified in 63/198 (31.82%). Plasmid-mediated quinolone resistance genes such as *QnrS1* (27/198, 13.64%)**,** *QnrB17* (25/198, 12.63%)**,** and *OXA-1* (49/198, 24.75%) were also observed. Several *SHV* variants were found, including *SHV-187* (67/198, 33.84%)**,** *SHV-120* (23/198, 11.62%)**,** *SHV-110* (15/198, 7.58%)**,** and *SHV-106* (14/198, 7.07%)**,** while other β-lactamases such as *OKP-B-17* (2/198, 1.01%) and *ACT-6* (2/198, 1.01%) were rare. Very low-frequency genes (each detected in one isolate; 0.51%) included *ErmB*, *SHV-1*, *OKP-B-5*, *FosB1*, *QepA2*, *LEN-26*, and others (see Fig. 4A). Gene profiling revealed that core AMR gene presence clustered together across isolates (see Fig. 5A). Among the 198 isolates, 196 exhibited a resistance score of 1, 1 isolate showed a score of 0, and 1 isolate, *K. pneumoniae* ST290-1LV SRR24046077 displayed a resistance score of 2 (see Fig. 5A).

### Capsular (K) and O-antigen serotyping

The analysis of the O-antigen and K-capsule loci revealed a predominance of specific O-locus and K-locus types among the 198 isolates. The most prevalent O-locus was OL2α.1, identified in 92 (46.5%) isolates, followed by OL2α.2 in 63 (31.8%) and OL3γ in 18 (9.1%). Other O-locus types, including OL3α/β (nine isolates), OL5 (13), OL4 (6), OL13 (5), and OL10, OL12, OL15, and OL2α.3 (each <2%), were less common. In terms of K-locus (capsular) types**,** KL24 was the most frequently observed, detected in 34 (17.2%) isolates, followed by KL25 in 20 (10.1%)**,** KL102 in 9 (4.5%)**,** and KL16**,** KL30**,** KL23, and KL149 each in 6–7 isolates (Fig. 2A).

### Mobilome profile

Plasmid replicons and MGEs in the 198 *Klebsiella* isolates revealed extensive diversity and distribution. Across all isolates, a total of 24 distinct plasmid replicon types were identified. The number of plasmid replicons per isolate ranged from 1 to 10 (Fig. 4E). The most prevalent replicon types included IncFII_1_pKP91 in 150 isolates (75.76%)**,** IncFIB(K)_1_Kpn3 in 143 (72.22%)**,** IncR_1 in 136 (68.69%)**,** and Col440I_1 in 107 (54.04%). Other frequently detected replicons included ColRNAI**_**1 (65 isolates, 32.83%), IncFIB(pKPHS1)_1 (34, 17.17%), IncFIB(Mar)_1_pNDM-Mar (35, 17.68%), and IncHI1B_1_pNDM-MAR (36, 18.18%) (see Fig. 4D).

**Fig 6:**
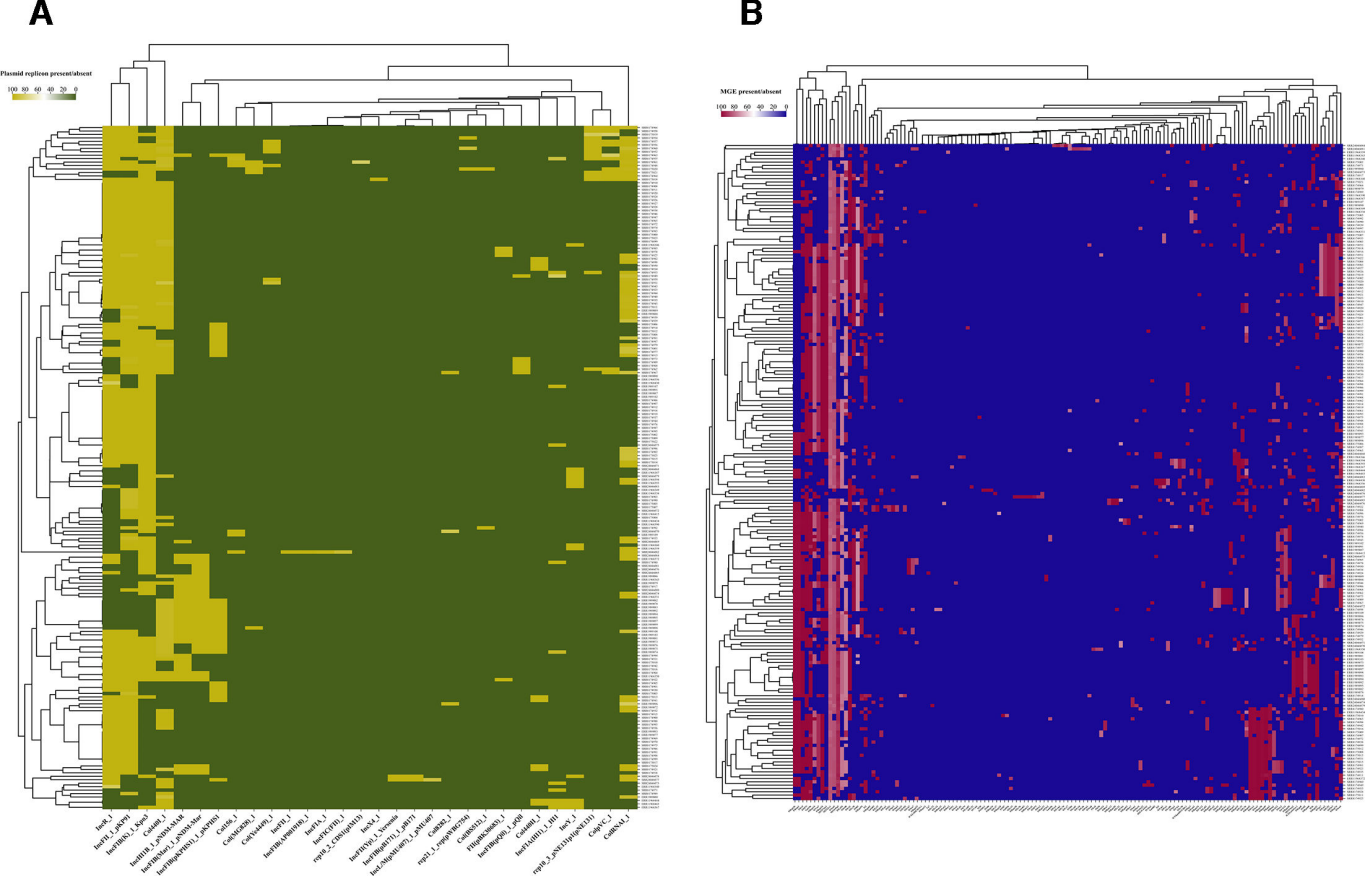
Plasmid replicon and MGE profiles. (**A**) Heatmap showing the presence or absence of plasmid replicons clustered by similarity. The heatmap color scale represents percent identity: green indicates absence or lower identity and yellow indicates presence at higher identity. (**B**) Heatmap showing the presence or absence of MGEs, including insertion sequences and integrative elements, clustered by similarity. The heatmap color scale represents percent identity: blue indicates absence or lower identity and dark red indicates presence at higher identity. Plasmid replicons and MGEs were detected using ABRicate (PlasmidFinder and ISfinder databases). Presence was defined as ≥90% sequence identity and ≥80% coverage. Clustering was performed using hierarchical clustering with Euclidean distance.

MGE profiling detected over 120 different MGEs, including insertion sequences, transposases, and integrative elements. The number of MGEs per isolate ranged from 2 to 28 (Fig. 4E). The most frequently observed elements included MITEYpe1 (194 isolates, 97.98%), MITEEc1 (175, 88.38%), IS5075 (151, 76.26%), ISEc9 (140, 70.71%), ISSen9 (148, 74.75%), and ISEcl10 (122, 61.62%). Additional common MGEs included ISKpn1 (108, 54.55%), ISKpn24 (105, 53.03%), and Tn6196 (101, 51.01%), while several low-frequency MGEs, such as ICEKpnHS11286-1**,** ISPlge3**,** and ISCfr12, were also detected in <10% of isolates (see Fig. 4D). The complete distribution of plasmid replicons and MGEs across isolates is visualized with heatmaps (see Fig. 6A and B).

## DISCUSSION

*Klebsiella pneumoniae* is rapidly evolving, with yet elusive virulome and resistome, posing a global threat to public health. Here, from comprehensive pangenome analyses, we report a multitude of STs with versatile virulome, resistome, and mobilome profiles reflecting a complex of pathogens with an open pangenome. Overall, the average assembly of 5.57 Mbp, GC content, and other genomic features indicate that most sequenced Tanzanian isolates have the average genome size of *K. pneumoniae* described from standard studies (2, 13, 14), and thus our downstream analyses and results suffice for genomic interpretation and inference.

From the pangenome architecture for 198 isolates, a high genome plasticity represented by a presence/absence matrix with gene families per isolate and a U-shaped frequency distribution in Fig. 1A and B is a hallmark of an open pangenome (11, 14). The persistence of 13.6% of the genes indicates that these genes could be present in ≥85% of genomes (core or nearly core) (Fig. 1B). This shows that these isolates comprise a minimal core genome, with the largest tailored to niche-specific functions or recent acquisitions. The shell genome of 11.2% of gene families indicated that the pangenome could be considerably conserved. The standard deviations of 0.21 for shell genes and 0.01 for cloud genes indicate moderate frequency variation among the strains (11). The cloud gene proportion of 75.2% suggests that most genes (3 out of 4) are found in <5% of genomes, indicating strong strain-to-strain variability and high horizontal gene transfer rates. These could be attributed to plasmids and mobile genetic elements, including phages (15). Overall, these findings suggest that the isolates in this work have a wide range of adaptations across hospitals, the community, and environmental reservoirs (13, 16). This underscores its adaptability in clinical settings, an evolutionary advantage posing risks of new AMR and complicating intervention initiatives.

As shown in Fig. 4, a versatile mobilome is represented by diverse plasmids, insertion sequences, and transposons of various families. The IncFII_1_pKP91, IncFIB(K)_1_Kpn3, IncR_1, ColRNAI_1, and IncFIB(pKPHS1) are the most frequently known plasmids within the KpSC, as also reported from other studies (17–19). Their occurrence has been attributed to the transmission of AMR genes conferring resistance to diverse β-lactams, aminoglycosides, sulfonamides, and macrolides, among other routine antibiotic classes (5, 18, 20, 21). Therefore, their prevalence in these Tanzanian strains presents a severe threat to the health system, not only because they can render *K. pneumoniae* untreatable but also present a scaffold for interspecific transmission within the family Enterobacteriaceae (3, 4, 16, 22).

We show that the distribution of MGEs is on a huge spectrum (Fig. 4B). This could account for the most possible agents of the observed pangenome plasticity as a result of high horizontal gene transfer rates leading to intra-strain and inter-strain genome diversity (14, 21). Although the study of mobile genetic elements is still at its natal stage globally, emphasis should be placed on the role of mobilomes in pathogen genome diversities and their epidemiological impacts for more effective outbreak surveillance and control strategies.

Most importantly, the SNP-based phylogeny in Fig. 2A clearly shows multiple subclades, which represent versatile lineages resulting from up to 683,177 high-quality core-genome SNPs. In addition to this huge number of SNPs and subclade clusters, we find overlaps among several STs, such as the cluster of ST45, ST39, ST336, and ST14. This indicates a huge diversity, which could be reflected in the virulome and resistome patterns associated with a variety of STs (23). From these findings, epidemiological studies focusing on *K. pneumoniae* are still needed to establish a conserved pattern of the core genome, which could help design broad-spectrum vaccines.

From our Kleborate analysis, the identification of 184 species shows that most of the KpSC species circulating in Tanzania comprise the *K. pneumoniae sensu stricto*. The recovery of 90 distinct STs from only four projects suggests a huge diversity of clones circulating in Tanzania. This suggests the need for comprehensive genomic surveillance studies to substantiate the risk of this diversity and predict future outbreaks for more informed interventions for the country.

We show that the prevalence of ST45 is the highest, accounting for over 10% of all 198 samples. The ST45 is a global clone, which has been implicated in neonatal sepsis with transmission of ESBLs and carbapenem resistance (18, 24).

Our results concur with these reports, demonstrating that most of the ST45 isolates are frequent in blood samples, representing an important risk of septic shock. Reported from Mwanza, the ST45 clone was also isolated from rectal swabs (18), suggesting that the clone can disseminate through multiple organs and systems. In addition, the ST45 has been shown to harbor the plasmid types IncFIB(K), IncQ, IncR, and/or IncFII(K)/IncR/IncFII(Yp) carrying genes encoding *aph(3′)-Ia*, *bleO*, *tet(A)*, and *dfrA14*, *bla_TEM1B_*, *bla_KPC-2_*, and *bla CTX-M-15*, conferring resistance to aminoglycosides, tetracyclines, fluoroquinolones, and third-generation cephalosporins (18, 24, 25). This clone has also been grouped into the 21 common *K. pneumoniae* lineages accounting for nosocomial transmission clusters, along with ST323 and ST340 (26). Therefore, given its high frequency and global distribution, the ST45 attracts attention as one of the most likely risks in the clinic and the environment, with the highest rate of AMRG transmission.

Among high-risk clones are ST11, ST307, ST37, ST348, ST101, ST14, ST147, and ST15, which have been characterized with MDR associated with nosocomial infections (18, 19, 27–29). Our findings, therefore, suggest a critical risk of MDR circulating in the country, with high-risk clones exacerbating the risk of ESBLs across the country. The next most prevalent clones, ST39 and ST336, and their public health risk have been determined by the ESBL gene *CTX-M-15* and carbapenem resistance genes (30). In Tanzania, Perdersen et al. (5) reported in 2020 that the occurrence of the ST39 clone, which harbors IncFII/IncR, was attributed to the dissemination of ESBLs, including *bla*CTX-M-15, among children in most hospitals. On the other hand, ST336 has been reported from other continents, including Australia and Europe, predominantly accounting for the *blaCTX*-M-15 form of ESBL in clinical and environmental settings (31, 32). The high prevalence of these clones could account for the corresponding prevalence of resistance to ceftriaxone, a third-generation cephalosporin of routine application in Tanzania (33). However, studies on AMR profiles have reported the prevalence of β-lactamases from clinical settings (3, 18, 34). Rigorous surveillance should combine targeting individual STs and genes with wet laboratory experimentation to come up with policy briefs suggesting reshuffles of the clinical AMR panels.

From virulence distribution results, the high frequencies of the siderophore genes *ybtA*, *ybtP*, *fyuA*, *irp2*, *ybtE*, *ybtQ*, *ybtS*, *ybtT*, *ybtU*, and *ybtX* suggest that 43% of the isolates possess this iron acquisition machinery. The gene *ybtA* encodes a yersiniabactin transcriptional regulator protein YbtA, which activates the expression of the yersiniabactin synthetase *irp2*, putative inner membrane ABC-transporter *ybtP*, and the iron acquisition outer membrane yersiniabactin receptor *psn/fyuA* (35), collectively required for synthesis and organization of yersiniabactin for uptake of iron from the environment. While the isolated ERR11968338 (ST348) harbors the aerobactin and colibactin clusters, SRR24046082 (ST307) possesses aerobactin in addition to the yersiniabactin cluster, therefore accounting for its highest Kleborate virulence score (Fig. 5). It is important to note that aerobactin and colibactin contribute to the potential for translocation from the gut to the bloodstream and systemic infection, as it has been commonly associated with meningitis (36, 37). In addition, colibactin has been shown to promote survival in hypervirulent hypermucoviscous *K. pneumoniae* (20, 38). Our K-PAM analysis confirmed that isolate SRR24046082, ST307, is potentially hypermucoviscous, carrying the gene *iucA*. Although the isolate lacks the regulator genes *rmpA* and *rmpA2*, evidence suggests that the possession of *iucA* is a sufficient factor for hypervirulence (39). As part of the BioProject PRJNA951629, the BioSample (SAMN34046999) indicates that the isolate is associated with bloodstream infections, supporting the inherent virulome of SRR24046082 (ST307). However, although this isolate has not been mentioned in the publication by the authors from the same project in Mwanza (3, 18), our analysis reports this isolate as one of the most important threats owing to its hypervirulence, not to mention its ESBLs. Interestingly, a study by Heiden and colleagues (40) recently reported an outbreak of the ST307 clone from Germany that demonstrated hypermucoviscous phenotypes. These findings strongly support our speculation that the ST307 isolate in this work could account for bloodstream and systemic infections. Overall, combining Kleborate with ABRicate/VFDB results, the most prominent component of the virulome belongs to the iron acquisition class, especially the yersiniabactin. These findings concur with those reported from an epidemiological genomic analysis study by Spadar and colleagues in 2022 from Portuguese hospitals (41).

On the other hand, information on K and O-antigen serotyping of *K. pneumoniae* in Tanzania is missing. This indicates that circulating *Klebsiella* clones are largely uncharacterized. Even in the projects from which we extracted our sequences (4, 5, 18), capsular polysaccharides and O-antigen typing were not reported. Here, through the application of Kleborate, we show that most of the isolates possess the locus O1 (OL2α.1), followed by O2 (subtype O2α.2L). Similar results have been reported from other countries, including Ghana (42), China (43), and Australia (26). Recently, in Uganda, MDR *K. pneumoniae* clones were also reported to predominantly possess the O1 and O2 loci in the same ranking (28). Therefore, our findings concur with other studies that most strains possess the O1 and O2 loci (5, 26, 28, 43), suggesting that vaccine strategies aiming to utilize the O1 and O2 antigens are likely to cover a wide range of strains. However, detailed analysis reveals intra-serotype variations, which suggest that there is a huge spectrum of serotype variants yet to be substantially elucidated. Evidence shows that serotypes O1 and O2ac provide an unusual mechanism for antigen diversification (44), potentially linked to different epitope variants. For instance, the O2 subtypes detected in Gorrie et al. (26) were O2afg, O2a, and O2ac, while in this study, we detected the O2α.2L, suggesting a need for experimental and downstream immunotyping to appropriately design relevant vaccine constructs. In addition, we note that O3 and O5 account for about 12% of all the isolates in this study, thus also attracting attention as an important category to consider for intervention. These findings are consistent with those from a multicounty study, which showed that O3 and O5 also comprise a significant group of up to 25% of all the isolates (45). Overall, combining our findings with those of others (26, 28, 43, 45), we recommend that vaccines designed for *K. pneumoniae* should consider O1, O2, O3, and O5 as priority serotypes along the intervention trajectory.

On the other hand, we observe a slightly distributed pattern of K types, although KL24 is the most prevalent (17.2%), followed by KL25 (10.1%). In this work, we observe in Fig. 2 that KL24 matches with OL2α. Considerable evidence shows that strains carrying the serotype KL24:O2a are highly virulent and strongly associated with systemic infections (46). These findings are partially consistent with the multicounty study by Choi et al. (45), who also observed that KL24 was the most common in their study by 7%. However, the next most common was KL2, which was completely not detected in all the isolates of the present study. In the Shanghai study by Wang et al. (43), the most common K loci were KL1 (54%), KL2 (18%), followed by KL24 and KL25 (9% each). These findings support the observation that KL24 and KL25 are common, although further research is necessary to characterize more local and international samples. In Uganda, Byarugaba and colleagues (28) found that the most common K type was KL3 (*n* = 7), but KL24 (*n* = 3) and KL25 (*n* = 3) were the third. These results reiterate that the K loci are versatile across the KpSC, strongly suggesting a need to embark on the polysaccharide K loci for better information leading to intervention design.

Generally, we note that the range of 6–68 AMR genes in Fig. 4E provides evidence that all the isolates are resistant to at least one antibiotic class. Without delving into mutations and SNP AMR mechanisms, it is sufficient to infer that most of the circulating clones possess acquired β-lactams, including *CTX-M-15* and *TEM-1*, representing ESBLs conferring resistance to cephalosporins and penicillins. Of importance, *OXA-* and *SHV-*gene variants observed in this work even worsen the burden of ESBLs in the country. These ARGs have been internationally attributed to the most common causes of medical emergencies and mortality, especially in low-resource settings (47, 48). Other very important ARGs are the multidrug transport protein *acrB* and the macrolide resistance gene *marA*. These, together with the aminoglycoside resistance genes *AAC(3)-IId* and *APH(3″)-Ib*, could account for reported antibiotic resistance cases in Tanzania (49, 50). Overall, the genome of *K. pneumoniae* ST290-1LV (SRR24046077), which demonstrates the highest resistance score as indicated in Fig. 5, harbors the gene variants *ACT-63*, *OXA-1*, *TEM-1D*, and *OXA-60*, which make it carbapenem resistant. The *ACT* genes, also known as *AmpC* β-lactamases, belong to the C β-lactamase class and have long been largely carried by the *Escherichia coli* and *K. pneumoniae*, as cephalosporinases conferring resistance to various β-lactams, that cannot be inhibited by clavulanic acid (51, 52). Which variants are eminent in *K. pneumoniae* may be a question of comprehensive investigation. Here, we show that the *ACT-63* variant is carried by one unique strain of ST290, representing a distinct AMR genotype, in addition to the highest AMR score. Moreover, evidence about the presence of the *OXA-60* gene variant in *K. pneumoniae* is lacking. However, available reports from France, Croatia, and Japan suggest that the *OXA-60* has been exclusively carried by *Ralstonia pickettii*, conferring resistance to multiple aminoglycosides, ticarcillin-clavulanate, aztreonam, and meropenem (53–55). Both ST290 and *R. pickettii* have been reported as common environmental and clinical nosocomial pathogens (56, 57), which could explain their potential for interspecific gene transfers. With the observed open genome structure, *K. pneumoniae* strains may have undergone an evolutionary transition accompanied by gain and/or loss of some AMR genes and/or mutations, given the fact that our present study covers genomes sequenced over a decade. However, to establish this trend, a retrospective longitudinal study is highly recommended, which could integrate genomic and antibiotic susceptibility test results with statistics and mathematical modeling of data sets from clinical and environmental studies.

### Conclusion

The diversity of the KpSC presents a complex repertoire of intraspecific differences associated with differential adaptive potentials across a wide range of environments. In this work, 198 isolates from Tanzania portray a huge pangenome plasticity, which largely comes from a spectrum of plasmids, insertion sequences, and transposons. Owing to these factors, the complex is versatile, presenting with high-risk international and pandemic clones including the most prevalent ST45, ST39, ST336, ST14, ST1552, and ST17 with KL24 and KL25 loci and OL2α.1 and OL2α.2 serotypes emerging the most prevalent. We eventually show that the overall virulome is largely determined by the yersiniabactin cluster, while the resistome is marked by resistance genes to virtually all routine antibiotics used in Tanzania, although the ESBL blaCTX-M-15 remains outstanding. Taken together, these findings infer that the KpSC species in Tanzania comprise an open pangenome, which calls for large-scale surveillance studies to customize interventions for effective control of epidemics.

## Data Availability

All genomic data analyzed in this study were obtained from the NCBI database and are publicly available. The accession numbers for these datasets are provided in the Supplementary Material.

## SUPPLEMENTAL MATERIAL

Supplemental tables spectrum.01947-25-s0001.xlsx Tables S1 to S3.

ASM does not own the copyrights to Supplemental Material that may be linked to, or accessed through, an article. The authors have granted ASM a non-exclusive, world-wide license to publish the Supplemental Material files. Please contact the corresponding author directly for reuse.
